# Novel tonometer device distinguishes brain stiffness in epilepsy surgery

**DOI:** 10.1038/s41598-020-77888-0

**Published:** 2020-12-01

**Authors:** Aria Fallah, Thirusivapragasam Subramaniam, H. Westley Phillips, Xavier Michalet, Harry V. Vinters, William H. Yong, Joyce Y. Wu, Noriko Salamon, Benjamin M. Ellingson, Anthony C. Wang, Samuel D. Reyes, George M. Ibrahim, Alexander G. Weil, Julia W. Chang, Diana Babayan, Jimmy C. Nguyen, Eric Behnke, Chi-Hong Tseng, Gary W. Mathern

**Affiliations:** 1 Department of Neurosurgery, David Geffen School of Medicine at UCLA, 300 Stein Plaza, Suite 525, Los Angeles, CA 90095-6901 USA; 2 Department of Pediatrics, David Geffen School of Medicine at UCLA, Los Angeles, USA; 3 Department of Chemistry and Biochemistry, UCLA, Los Angeles, USA; 4 Department of Pathology and Laboratory Medicine, David Geffen School of Medicine at UCLA, Los Angeles, USA; 5 Division of Pediatric Neurology, David Geffen School of Medicine at UCLA, Los Angeles, USA; 6 Department of Radiological Sciences, David Geffen School of Medicine at UCLA, Los Angeles, USA; 7 Division of Neurosurgery, The Hospital for Sick Children, University of Toronto, Toronto, Canada; 8 Division of Neurosurgery, Ste. Justine Hospital, University of Montreal, Montreal, Canada; 9 Department of Clinical Neurophysiology, David Geffen School of Medicine at UCLA, Los Angeles, USA; 10 Department of Medicine, David Geffen School of Medicine at UCLA, Los Angeles, USA; 11 Department of Psychiatry and Biobehavioral Medicine, David Geffen School of Medicine at UCLA, Los Angeles, USA

**Keywords:** Epilepsy, Neural circuits

## Abstract

Complete surgical resection of abnormal brain tissue is the most important predictor of seizure freedom following surgery for cortical dysplasia. While lesional tissue is often visually indiscernible from normal brain, anecdotally, it is subjectively stiffer. We report the first experience of the use of a digital tonometer to understand the biomechanical properties of epilepsy tissue and to guide the conduct of epilepsy surgery. Consecutive epilepsy surgery patients (n = 24) from UCLA Mattel Children’s Hospital were recruited to undergo intraoperative brain tonometry at the time of open craniotomy for epilepsy surgery. Brain stiffness measurements were corrected with abnormalities on neuroimaging and histopathology using mixed-effects multivariable linear regression. We collected 249 measurements across 30 operations involving 24 patients through the pediatric epilepsy surgery program at UCLA Mattel Children’s Hospital. On multivariable mixed-effects regression, brain stiffness was significantly associated with the presence of MRI lesion (β = 32.3, 95%CI 16.3–48.2; *p* < 0.001), severity of cortical disorganization (β = 19.8, 95%CI 9.4–30.2; *p* = 0.001), and recent subdural grid implantation (β = 42.8, 95%CI 11.8–73.8; *p* = 0.009). Brain tonometry offers the potential of real-time intraoperative feedback to identify abnormal brain tissue with millimeter spatial resolution. We present the first experience with this novel intraoperative tool for the conduct of epilepsy surgery. A carefully designed prospective study is required to elucidate whether the clinical application of brain tonometry during resective procedures could guide the area of resection and improve seizure outcomes.

## Introduction

The most common cause, and arguably the most challenging substrate of drug-resistant epilepsy in children, is cortical dysplasia (CD)^1–3^. Surgical treatment depends on the ability of the preoperative investigations to determine a spatially well-defined focus for resection. It is particularly challenging to adequately identify and resect the full extent of CD for several reasons^3^: First, magnetic resonance imaging (MRI) may not visualize the entirety of the lesion (“Tip of the iceberg phenomenon”) or may be altogether “non-lesional”^4–6^. Second, electrocorticography (ECoG) to determine the extent of CD is limited by issues related to spatial coverage, analysis protocols and biomarker selection^7^. Finally, CD appears identical to more normal brain during surgery, making intraoperative determination of the surgical borders difficult.

Despite the use of an intraoperative microscope, neurosurgeons cannot often visually differentiate normal tissue from CD, especially if the CD is subtle (i.e. Type I). However, some neurosurgeons palpate tissue stiffness to identify the boundaries of dysplastic tissue specially after the arachnoid is transgressed. Complete surgical resection of the structural lesion has been repeatedly shown to be the most important, and often the only, predictor of seizure freedom after epilepsy neurosurgery^8–16^. Complete resection is associated with 70–87% probability of seizure freedom while an incomplete resection is associated with 5–49% probability of seizure freedom^10^. Surgical failures leading to seizure recurrences are costly, often require additional surgery, and are associated with continued elevated risks of seizure-related mortality and morbidity compared to those who achieve seizure remission^17,18^. Novel biomarkers to assist in the conduct of epilepsy surgery are welcome, yet very little innovation in surgical technique has been realized in the past 80 years.

Here, we investigate the utility of intraoperative real-time tonometry in identifying pathological tissue on the basis of brain stiffness. Disease-related changes in tissue stiffness are common in medicine^19–21^ including a variety of neurological disorders^22,23^. Epilepsy surgeons also qualitatively use tactile feedback to differentiate normal brain tissue from CD to guide the boundaries of surgical resection. Through this study, we investigate a novel application of a digital tonometer to in vivo human brain tissue and correlate the findings to imaging and histopathological abnormalities. The findings of this study should inform the conduct of a larger, prospective study to determine whether brain stiffness could be leveraged to improve outcomes for respective epilepsy surgery.

## Methods

Consecutive patients identified for resective epilepsy surgery at UCLA Mattel Children’s Hospital who met the eligibility criteria were invited to participate in this prospective observational study. All patients met ILAE definition of drug resistant epilepsy^24^. Two authors, A.F. and T.S., reviewed the relevant clinical history, results of non-invasive testing (including non-invasive video-EEG, 3-T MRI, Fludeoxyglucose (FDG)-Positron Emission Tomography (PET) and magnetoencephalography (MEG), when available) and pre-planned approximately 10 total points on the lateral surface of the brain that would be exposed during the surgery from both presumed more normal and more affected regions. The Institutional Review Board (IRB) at the University of California–Los Angeles (UCLA) approved all experimental protocols, the participation of human subjects (IRB#17-001669) and all methods were carried out in accordance with these standard guidelines and regulations. Informed consent was obtained from all participants or their families including University of California HIPPA Research Authorization for Release of Personal Health Information for Research. We prepared a protocol a priori but did not register it.

### Clinical data extraction

We recorded age at seizure onset, gender, race, number of anti-seizure drugs at time of surgery, age at surgery, presumed or histologically confirmed diagnosis, and the results of video EEG, MRI, FDG-PET, and MEG. We also recorded the severity of histological changes (dichotomized into mild cortical disorganization and Chaslin’s gliosis vs. moderate or severe cortical dysplasia and other pathologies). To account for possible confounding variables, we also recorded side of surgery as well as physiological parameters including heart rate, systolic blood pressure, diastolic blood pressure, and arterial blood oxygenation at the time when brain stiffness was measured.

### Eligibility criteria

*Inclusion Criteria*

- Participants eligible for surgery through the pediatric epilepsy program at UCLA Mattel Children’s Hospital and/or operated on by the lead surgeon (AF).
- Undergoing resective epilepsy surgery for dysplastic (e.g., CD, TSC, Hemimegalencephaly, polymicrogyria) or non-dysplastic etiology (e.g., developmental tumors, gliosis, stroke, Rasmussen’s encephalitis, Sturge-Weber Syndrome).

*Exclusion Criteria*

- Lesion of interest is in difficult-to-access regions such as paralimbic structures, insular, depth of sulcus, or interhemispheric locations.

### Digital tonometer used to measure brain stiffness

**Figure 1:**
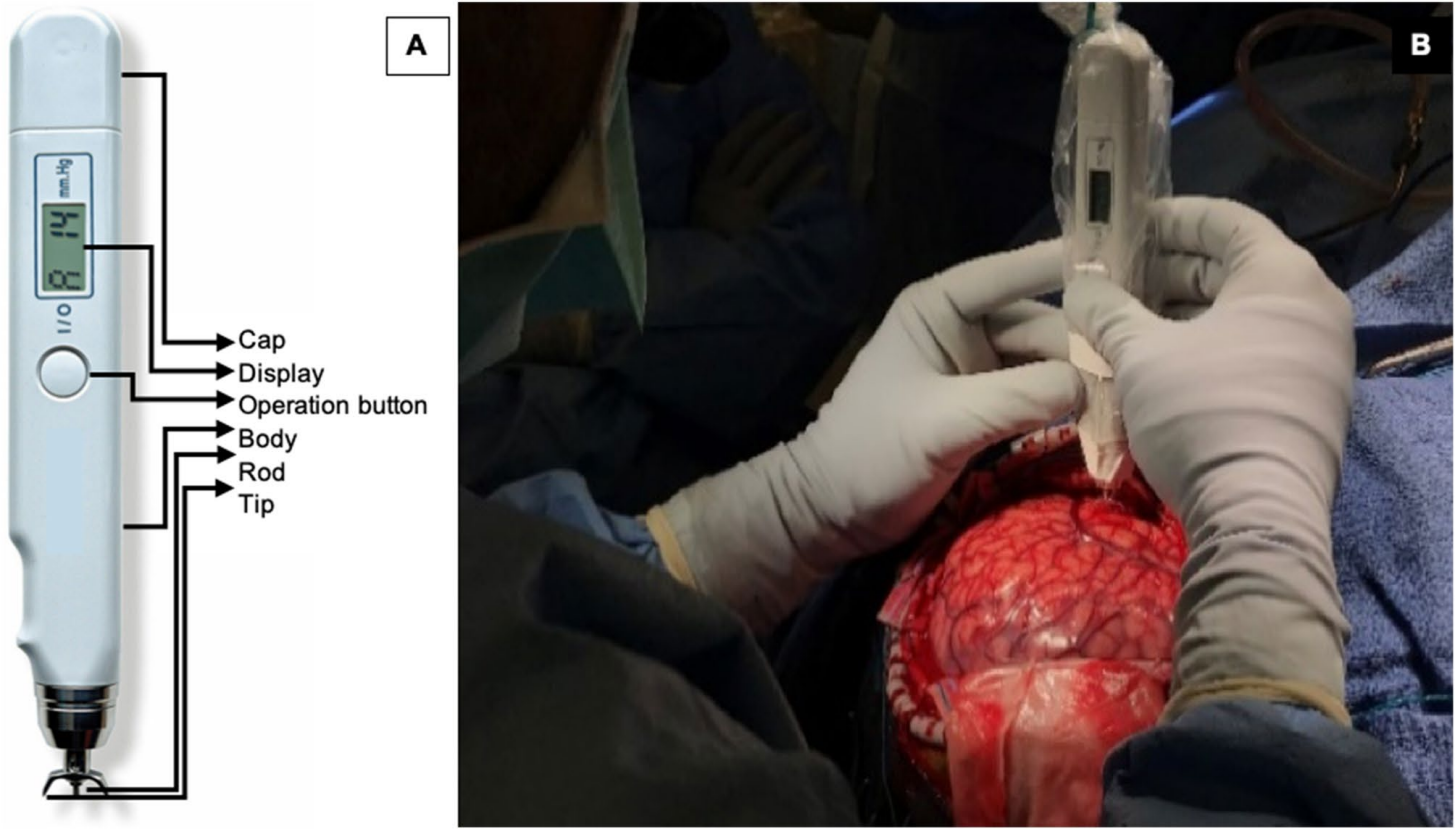
(**A**) The components of the digital tonometer (only the center rod and crescent-shaped tip make contact with the brain); and (**B**) intraoperative photograph demonstrating its use.

The Diaton Tonometer probe is simple to use and reliable. It directly measures cortical stiffness without obscuring the surgical field. Its metal components are sterilizable via the autoclave. Only minimal contact is made with the gyrus of interest involving the tip and rod, minimizing contamination risk while providing high-precision (millimeter) spatial resolution (Fig. 1). This degree of precision allows accurate comparisons to be made with histopathologic analysis. The measured pressure (P) is not the same as Young's modulus (E), and therefore cannot be compared directly to the literature values for Young's modulus of brain tissues. It is true that E is related to P in a non-linear fashion and that higher P means greater stiffness, but the exact relationship between these two variables is yet to be investigated.

### Our technique to obtain brain tonometry readings

Following the craniotomy, the dura is reflected facilitating a wide exposure to the lateral surface of the neocortex. Prior to ECoG, we perform brain tonometry on the crown of the gyri using the digital tonometer, which is approved by the U.S. Food and Drug Administration to measure intraocular pressure. The digital tonometer probe’s body is disinfected and inserted in a sterile plastic bag. The sterilized metal components are then attached to the probe through a small slit in the plastic drape. The device provides a digital readout of “pressure” and has a guaranteed maximum measurement error of 2 mm Hg in the range 5–20 mm Hg and of 10% in the range 20–60 mm Hg. The device’s principle consists in measuring the maximum deformation *d* of the tissue impacted by a small free-falling metal rod of mass *m*. Given the known surface area *S* of the contact zone, the reported pressure *P* is given by *P* = *m* × *g/S*, where *g* is the acceleration of the rod due to gravity and *S* is related to the deformation *d*. The reported pressure can then in principle be related to Young’s modulus *E* of the tissue (assumed to be isotropic), a direct measurement of its stiffness.

### Selecting regions for brain tonometry

We obtained stiffness measurements in pre-planned presumed more affected and less affected areas of the brain based on criteria stated previously^25^. We also obtained measurements from distant brain locations including recordings from each exposed lobe (Fig. 1). For each reading, we saved the stereotactic point onto our frameless neuronavigation software to facilitate its correlation to MRI and FDG-PET abnormalities. On average, we obtained 8 to 10 points per surgery. Each digital readout on the tonometer is an average of 4 to 6 individual pressure readings in millimeters of mercury (mm Hg). The device audibly alerts the operator when each measurement is consistent with the previous, therefore signifying high reproducibility of the readings. If the planned resection involved regions were brain stiffness was measured, we sent separate biopsies of these regions for histological analysis. We anonymously labeled these brain regions (such as brain region 1) to avoid inadvertently influencing the diagnosis. Brain stiffness measurements were all performed by the operating surgeon, (A.F.) and transcribed during surgery by the research fellow, T.S.

### Design considerations and statistical methods

It is important to note that there are no normal controls for this type of research. Post-mortem brain tissue undergoes irreversible biomechanical tissue changes, and there are no normal patients that undergo neurosurgical operations that might provide a good comparison. To address this issue, we use an experimental design that involves both within-participant, and between-participant comparisons. For continuous data, we report means and standard deviations. For dichotomous outcomes, we report frequencies and percentages. Means and standard deviations (SD) for brain stiffness measures are compared for each categorical covariate by a T-test or ANOVA (when there were more than 2 categories). We reported our findings using a coefficient (β), 95% Confidence Intervals (CI) and p values. To better account for the clusters of related data from each participant, we performed a linear mixed effects model with stepwise forward variable selection to identify covariates that independently predict brain stiffness. We created boxplots to compare the stiffness distribution by pathology and severity of histopathological abnormality. Receiver operating characteristic (ROC) analysis was performed to evaluate brain stiffness as a biomarker for underlying MRI abnormality, FDG-PET hypometabolism, and severity of histopathological abnormality, respectively. By conventional criteria, we considered results statistically significant if two-sided p values were less than 0.05. All statistical analyses were performed using IBM SPSS Version 25; IBM Corp.

## Results

We attempted brain stiffness recordings 31 times across 25 consecutive participants (Median age at surgery was 12 years; Range was 1–64 years) and obtained 249 data points (Table 1). The device malfunctioned due to blood contamination of the rod preventing measurements for participant #5 leaving 24 participants that contributed data to this study. The median age of seizure onset was 2 years (Range 0.11–64 years). Forty-five percent (n = 11) of the participants were female. The median duration of seizures was 4 years (Range 4 days to 15 years). From 30 total operations, 1 was for an anatomic hemispherectomy, 4 were for a functional hemispherectomy, 15 were for a cortical resection, 1 was for a cortical resection plus insertion of a Responsive Neurostimulation (RNS) device, 3 were for implantation of an RNS device only, and 6 were for invasive-electroencephalography (subdural grid strip and depth electrodes) alone. Three patients (13%) have had previous craniotomies. The underlying epileptogenic substrate was CD for 14 patients (58%), tumors for 3 patients (13%), infarction for 3 patients (13%), TSC for 3 patients (13%) and Rasmussen’s Encephalitis for 1 patient (4%). For 3 cases, we presumed a diagnosis of CD although no pathological specimen was available for confirmation; these patients underwent an RNS device implantation only. No infection or side effect was recorded in any patient due to brain tonometry. We estimate that our study protocol added 30 min to the surgical time although this was not prospectively recorded.

**Table 1:** Characteristics of participants included in the study.

Pt. no.	Age at surgery /gender	Race	Age of seizure onset	# of AEDs	Histopathological diagnosis	EEG localization /lateralization	MRI abnormality	FDG-PET (hypometabolism)
1	3y M*	Caucasian	8 m	1	CD Ic	Right T	Polymicrogyria	R hemispheric
2	4y M*	Hispanic	6 w	2	Tuber	Right F,T,C	Multiple cortical and subcortical tubers	Multiple bilateral foci
3	15y F	Middle Eastern	40 d	2	CD II	Left C,T,F	T2/FLAIR hyperintensity in left mesial and anterior temporal lobe	L Temporal lobe
4	3y F	Caucasian	9 m	2	Remote infarction and gliosis	Left H	Chronic infarction in L MCA territory	L MCA territory
5	2y F	Caucasian	16 m	1	Ganglioglioma WHO Grade I, CD IIIb	Left C	T2 hyperintensity in L amygdala and mesial temporal lobe	Left anteromedial Temporal Lobe
6	5y F	Hispanic	2 y	4	CD IIa	Bifrontal	FLAIR hyperintensity in R orbitofrontal region	Symmetric cerebellar
7	6y M	Mixed	4 m	2	Ulegyria and CD IIId	Left P	Restricted diffusion within cortex of L Parietal and Occipital Lobe	L Temporal, Parietal, and Occipital Lobes
8	20y M	Caucasian	5 y	4	Gliosis	Bilateral F,T	Subtle GW differentiation in L Temporal pole	L Temporal Lobe
9	22y F	Caucasian	11 y	3	CD**	Right C,T,P	Tiny foci of T2/FLAIR L periventricular hyperintensity	None
10	4y F	Black	4 y	1	Angiocentric Glioma WHO Grade I	Left F,T,C	Cortically based mass in L frontal operculum	L Temporal Lobe
11	12y M	Caucasian	2 y	3	CD**	Left C	None	None
12	14y M	Caucasian	7 y	2	CD**	Left T	T2/FLAIR hyperintensity in L Temporal Lobe	R greater than L Temporal Lobe
13	11y F	Hispanic	3 m	3	CD IIa	Left F	Residual FCD L anterior insula	None
14	64y F	Asian	1 m	3	Acute Infarction**	Diffuse	R Frontal Infarction	R Frontal Lobe
15	4y M	Caucasian	4 d	3	Remote Infarction	Right H	R hemispheric encephalomalacia	R Frontal and Temporal Lobe
16	13y M	Hispanic	1 m	3	CD IIa	Right H	Cortical thickening of R Frontal Lobe	R Posterior Temporal and Inferior Parietal
17	10 m M	Middle Eastern	6 m	3	CD IIa	Non-lateralizing	Normal	R Occipital Lobe
18	11y M	Hispanic	10 y	2	CD IIb	Right F,T,P	R Temporal Lobe Lesion	R Temporal Lobe
19	15y F	Caucasian	3 m	1	Ganglioglioma and CD IIIb	Right T	L Temporal lobe mass	L Temporal Lobe
20	15 m F	Hispanic	1 m	4	CD IIa	Right T	R temporo-parietal dysplasia	R Temporal and Occipital Lobes
21	8y F	Caucasian	3 m	5	CD Ic	Left H	Remote L functional hemispherectomy	R hemisphere, L Temporal and Parietal Lobes
22	15 m F	Asian	3 m	2	Tuber	Right F,C	Multiple cortical and subcortical tubers	Multiple bilateral foci
23	2y M	Hispanic	2 m	3	CD IIb	Right T,P,O	Right parieto-occipital dysplasia	R Parietal and Occipital Lobes
24	15 m M	Hispanic	3 m	4	Tuber	Right F	Multiple cortical and subcortical tubers	R Frontal Lobe
25	3y F	Hispanic	36 m	4	Rasmussen’s Encephalitis	Right F,C	R peri-sylvian and frontal lobe atrophy	R hemisphere

*F* Frontal, *C* Central, *H* Hemisphere, *T* Temporal, *P* Parietal.

*Participant had a prior craniotomy.

**Presumed diagnosis (No pathological specimen).

**Figure 2:**
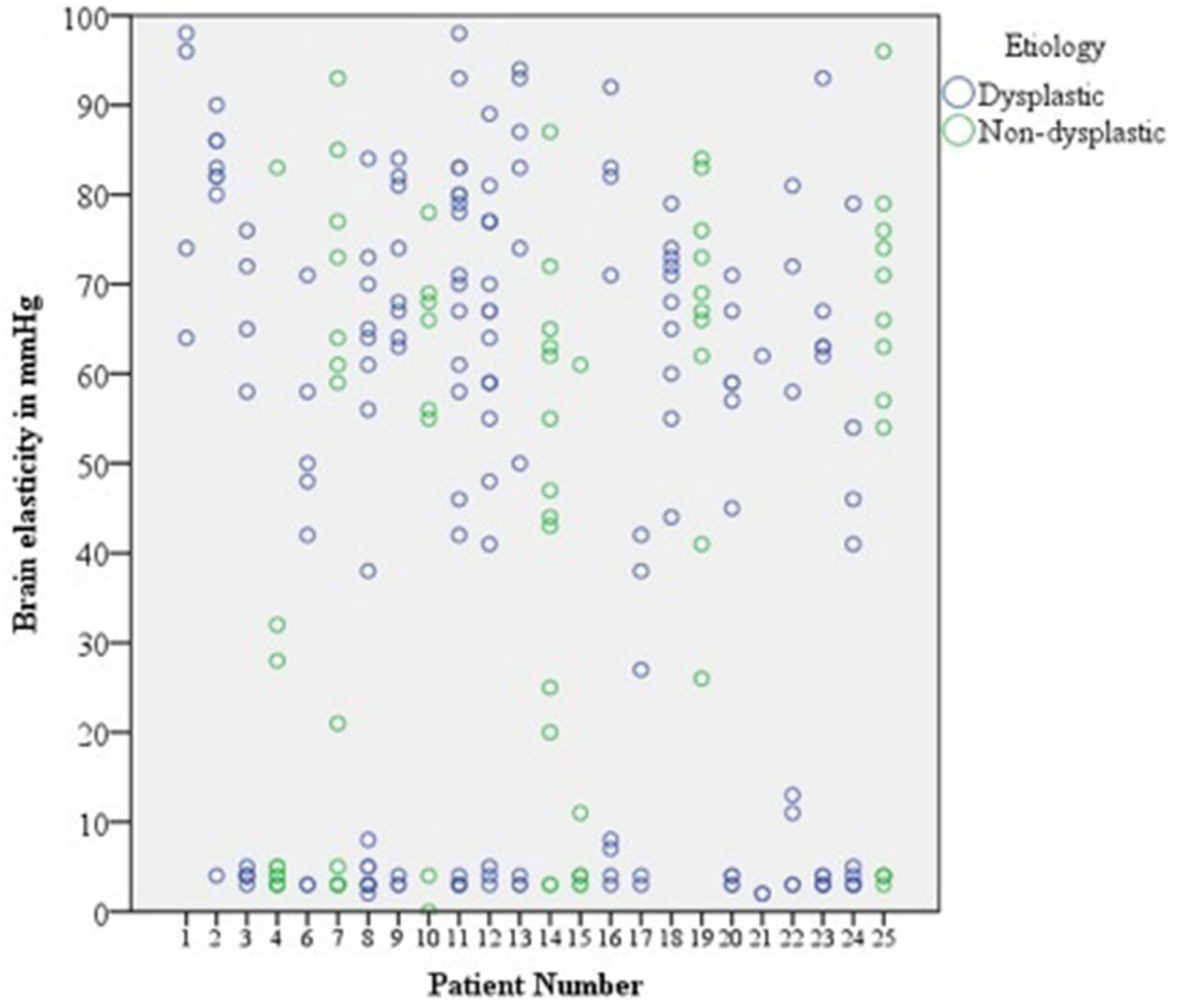
Scatter plot of raw data across 24 patients demonstrating a wide variability in brain stiffness measurements within participants.

**Figure 3:**
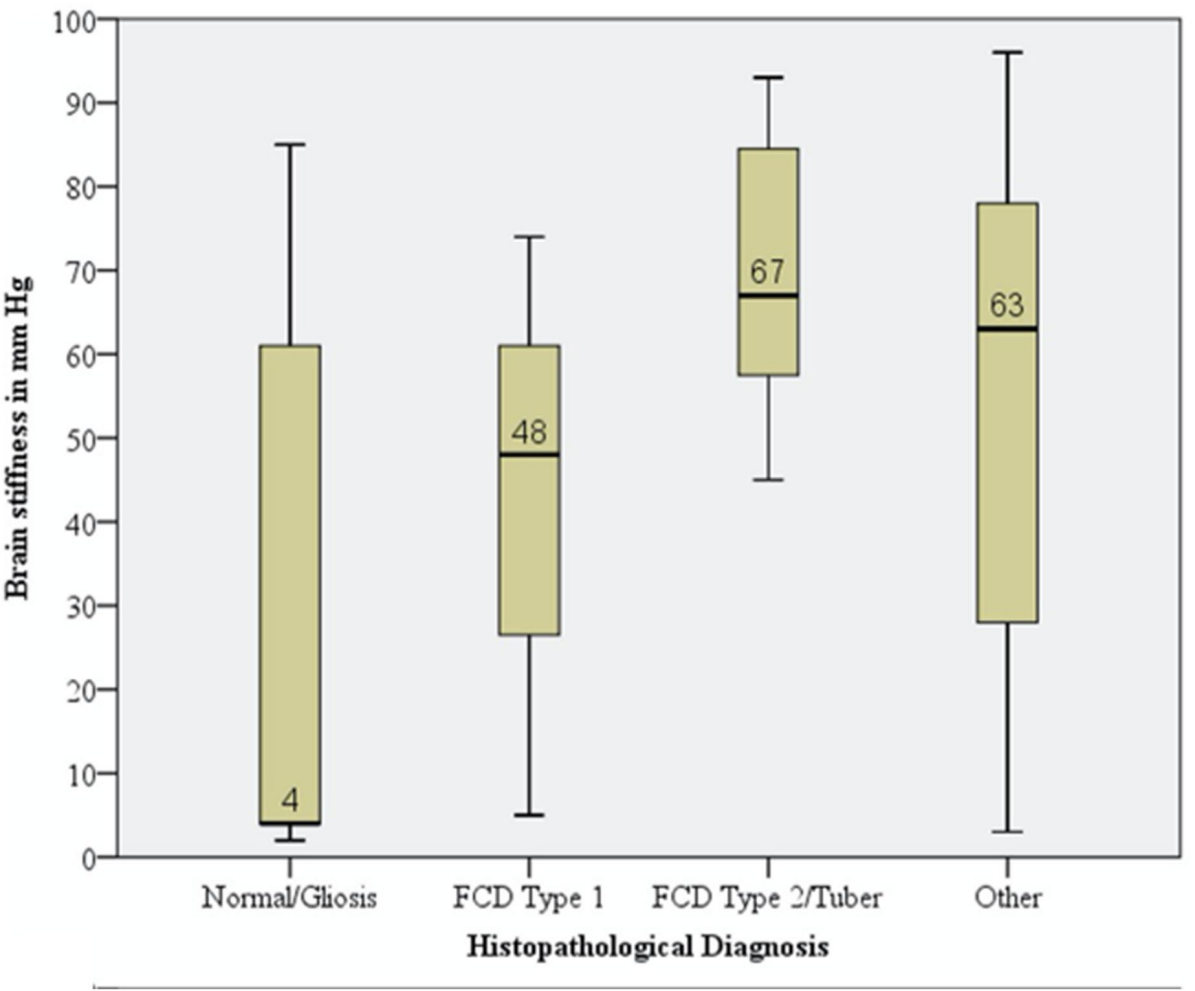
Boxplot of brain stiffness demonstrating mean, standards deviation and range in mm Hg by histopathological diagnosis.

Wide variability in effective brain stiffness was observed within individual participants (Fig. 2). In this cohort, the median brain elasticity was 55.0 mm Hg while the standard deviation was 33.2 mm Hg. In all patients, except 1, 18 and 19, we consistently found a tight cluster of measurements with low stiffness (Mode: 3 mm Hg; Mean (SD) = 3.6 (1.0) mm Hg). We presume this to correspond to more normal areas of the brain. In instances where this region was biopsied, this corresponded to mild cortical disorganization or Chaslin’s gliosis (Fig. 3).

Through univariable analysis, we found several covariates associated with higher brain stiffness including age of seizure onset (greater than 2 years), duration of seizures (greater than 7 years, prior craniotomy, recent subdural grid implantation, lesional MRI, and severity of cortical disorganization (Table 2). These covariates, all with p values of less than 0.20, were selected for multivariable hierarchical forward stepwise regression. A significant regression equation was found (F(4,32) = 18.268; *p* < 0.001), with an adjusted *R*^2^ of 0.683 (Standard Error (SE) 18.961). Presence of MRI lesion (β = 32.3, 95%CI 16.3–48.2; *p* < 0.001), severity of cortical disorganization (β = 19.8, 95%CI 9.4–30.2; *p* = 0.001), and recent subdural grid (β = 42.8, 95%CI 11.8–73.8; *p* = 0.009) were all independent predictors of brain stiffness. The high adjusted regression model fit *R*^2^ value suggests a high predictive ability of brain stiffness using the above variables. Of note, the presence of MRI lesion and severity of cortical disorganization together explained greater than half the variance of brain stiffness (adjusted *R*^2^ of 0.607 (SE 21.124)).

**Table 2:** Brain elasticity measures by covariates. T-test performed for dichotomized variables. ANOVA test performed when variables had more than 2 categories.

Independent variable	Mean (SD) mm Hg		Mean (SD) mm Hg		*p* value
Age of seizure onset (dichotomized)	< 2y 36.0 (35.0)		≥ 2y 49.8 (30.0)		< 0.001**
Duration of seizures (dichotomized)	< 7y 42.3 (32.3)		≥ 7y 44.6 (34.5)		0.110**
No. of AEDs (dichotomized)	< 2 50.0 ( 31.9)		≥ 2 42.3 ( 33.3)		0.230
Age at surgery (dichotomized)	< 12y 40.7 ( 33.7)		≥ 12y 46.3 (32.3)		0.211
Gender	Female 41.8 (32.2)		Male 44.5 (34.0)		0.517
Prior Craniotomy	No 41.0 (32.6)		Yes 56.0 (33.7)		0.011**
Side of Brain	Left 43.2 (33.7)		Right 43.3 (32.8)		0.976
Lobe	Frontal 47.6 (35.1)	Temporal 39.7 (30.6)	Parietal 40.6 (31.4)	Occipital 25.3 (40.6)	0.238
Post invasive EEG implant (grid, strip or depth)	No 40.7 (33.1)		Yes 56.0 (30.5)		0.007**
MRI	No Lesion 30.6 (33.0)		Lesion 54.5 (31.9)		0.058**
FDG-PET	Isometabolic 41.8 (33.2)		Hypometabolic 48.1 (32.9)		0.615
Diagnosis	Non-dysplastic 40.3 (32.2)		Dysplastic 44.5 (33.6)		0.610
Pathology	Normal/Gliosis 26.4 (32.4)	CD I 42.3 (34.8)	CD II/Tuber 69.7 (16.0)	Other 50.6 (34.9)	0.009**
Pathology (dichotomized)	Less affected 23.6 (31.4)		More affected 63.9 (23.1)		0.034**
Histopathological Severity	Mild 23.8 (31.3)	Moderate 67.7 (17.5)	Severe 73.0 (13.8)		< 0.001**

*For continuous variables, the cut-point was set at the median value.

**Statistically significant association with brain elasticity on univariate analysis. These variables were used for the multi-variate analysis.

**Figure 4:**
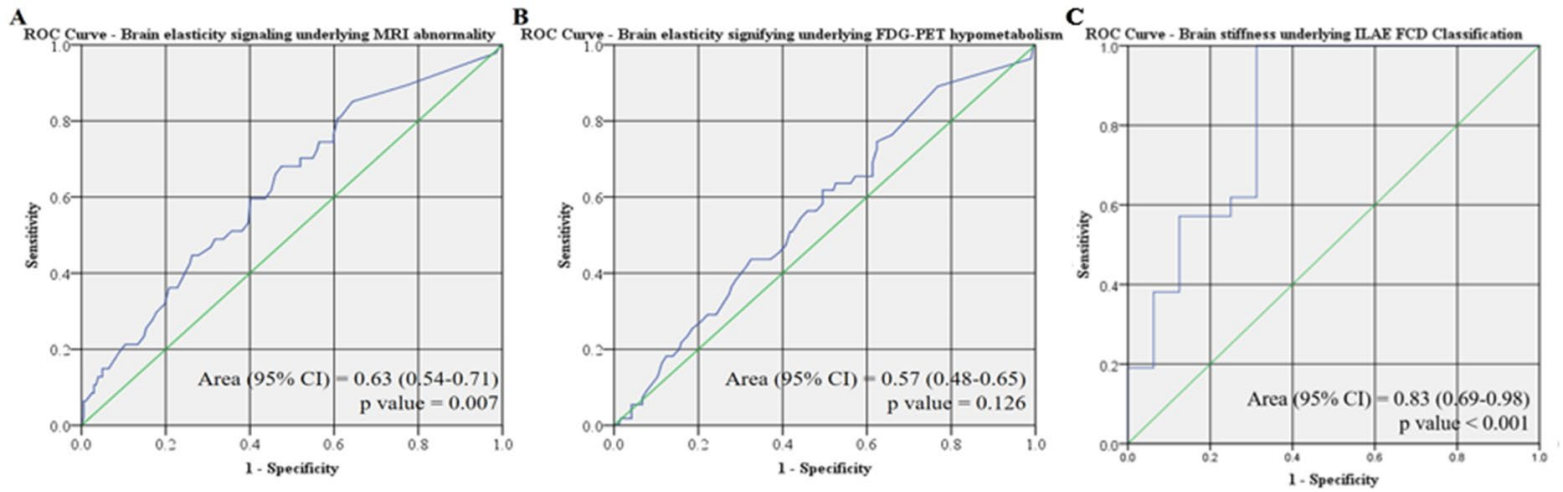
Receiver operator curve for brain tonometry as a diagnostic tool for detection of underlying (**A**) MRI abnormality, (**B**) FDG-PET hypometabolism; and (**C**) ILAE FCD Classification (normal/gliosis vs. Type 1/Taype 2 FCD).

For determining the utility of brain stiffness identifying underlying MRI lesion, FDG-PET hypometabolism and severity of histopathological changes (dichotomized to mild vs. moderate/severe cortical disorganization), we created a ROC plot for each imaging modality and obtained an area under the curve (AUC) of 0.63 (95% CI 0.54–0.71; *p* = 0.007), 0.57 (95% CI 0.48–0.65; *p* = 0.126) and 0.85 (95% CI 0.72–0.98; *p* < 0.001), respectively (Fig. 4). This analysis suggests that brain stiffness is a better biomarker for structural brain abnormalities than functional brain abnormalities based on traditional neuroimaging modalities. With a cutoff value of 60.5 mm Hg, there is 60% sensitivity and 60% specificity in detecting the presence of an underlying MRI lesion. Brain stiffness values have the greatest utility to serve as a biomarker for determining the severity of histopathological abnormality of the underlying neocortex. With a cutoff value of 16.5 mm Hg, there is 100% sensitivity and 71% specificity in detecting moderate/severe cortical disorganization from mild cortical disorganization.

## Discussion

**Figure 5:**
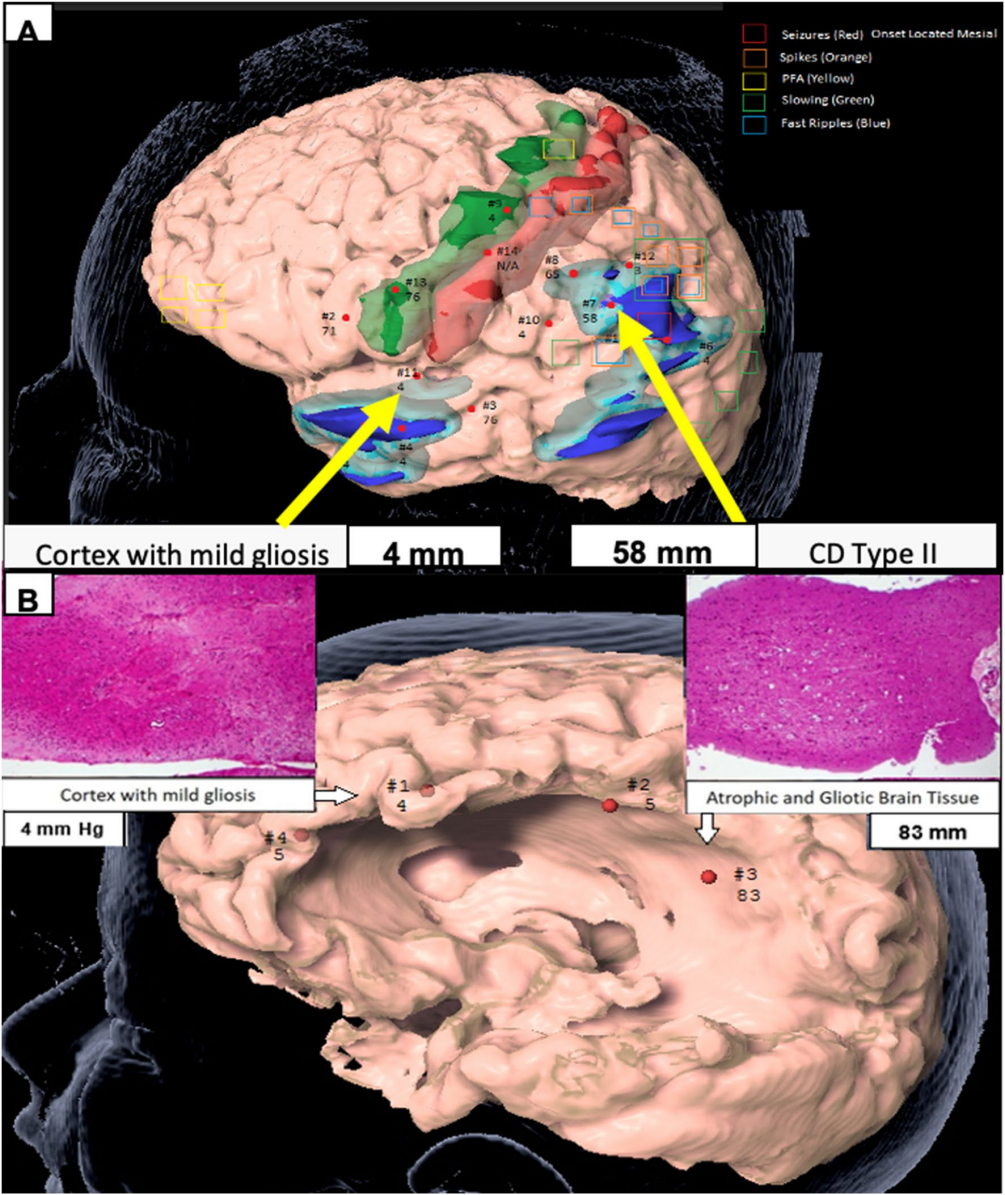
(**A**) Participant #3. Region of high stiffness corresponded to CD Type II while region of low stiffness corresponded to cortex with mild gliosis. Green is primary motor cortex. Red is primary sensory cortex. Light and dark blue are regions of PET hypometabolism and different thresholds. Legend describes the EEG data from the subdural grid and strip electrodes, and (**B**) participant #4. MRI biopsy location from a region of low stiffness (left) compared to an area of high stiffness (right) as measured using digital tonometry, and corresponding histopathology (H&E, magnification × 10). Figure designed using BrainLab iPlan Net 3.6.0 https://www.brainlab.com/radiosurgery-products/iplan-rt-treatment-planning-software/.

To our knowledge this is the first study to measure in vivo brain stiffness in epilepsy surgery patients. We report several novel findings. First, there is a large degree of variability in regional brain stiffness within participants. In participant 3 for example, this variability was demonstrated as adjacent gyri can have striking differences in stiffness (e.g., see point 7 and 12) (Fig. 5). Our data suggest that more than half of this variance is explained by histopathological severity and presence of MRI lesion alone. Dysplastic lesions induce changes in gene expression that alter molecular and cellular constituents in structurally normal brain regions^26^, perhaps secondarily affecting brain stiffness.

Second, regardless of the participant’s age at surgery, race, gender, epilepsy substrate, and lobe, there is a very tight range of normal brain stiffness corresponding to about 3 mm Hg on this device. Given its consistency, we believe that this likely reflects the stiffness of normal brain. Our study is similar to the findings reported by Chauvet et al. who found lesional tissue to have higher stiffness compares to more normal brain^27^. A direct comparison of the values is not possible at this stage as the two methods do not measure the same variable.

Third, we observed that for participants with CDs, and especially for Type I, they commonly had widespread abnormalities in brain stiffness even at distant sites (with no MRI or FDG-PET abnormality) from the resected seizure-onset zone. Taylor et al. suggested that epileptogenic CD is often distributed and may be not-contiguous^28^. Even within the confines of a single resected lobe, the abnormality is sometimes disseminated rather than confined to a single region. The findings of our study support this notion, which can be further tested through a biopsy of these regions in future studies.

### Prior studies on human brain stiffness

There are a number of in vivo and ex vivo studies on brain stiffness in animals^29^ and of ex vivo studies in humans, but we are aware of only two research teams that have performed in vivo direct measurements of human brain stiffness^30,31^. The first group applied a light aspiration device to the surface of an adult patient’s brain with a brain tumor and used an adjacent mirror that reflected the image to an external camera to measure the deformation of this tissue^32^. Although the primary aim was to assess applied pressure by synchronizing the video and pressure recording, the authors reported that this was a difficult experiment to perform with concerns regarding measurement reproducibility and challenges with sterilization. Another group published on the utilization of an ultrafast ultrasonic device to measure shear wave electrography as a means to characterize brain tumor subtypes and differentiate this from the surrounding normal brain^38^. This device obscures the surgical field, has a limited field of view and provides low-spatial resolution. Further, it does not provide immediate quantitative feedback to the surgeon. Other studies of brain stiffness are reported using non-invasive MR-based techniques such as MR Elastography (MRE) with no objective intraoperative verification^21,23,32^. Post-mortem and animal studies^33,34^ are not necessarily comparable to *in-vivo* studies in the human pediatric brain.

### Mechanism of variation in human brain stiffness

We postulate that the mechanistic origin of variations in stiffness correlates to underlying tissue microstructure and is altered by the following: (1) Individual neurons: Increased neuronal density is associated with increased tissue stiffness^35,36^; (2) The Extracellular Matrix: Increased tissue acidity, as a result of increased CO_2_ concentrations, is associated with increased stiffness^37^; and (3) The cellular cytoskeleton and other structure forming proteins: The human brain undergoes significant alterations especially over the first 4 years of life in which neurons exhibit more extensive dendritic and axonal branching and synapse formation accompanied by a rise in the lipid content as axonal segments are myelinated^38^. Increased myelin content increases stiffness, perhaps due to its high lipid content, leaving less space for the polymeric/structure-forming material such as collagen, actin, and tubulin^37^.

### Potential clinical implications

In less-well localized epilepsy, certain pediatric epilepsy groups, including ours, frequently determine surgical resection boundaries by avoiding eloquent brain regions. The argument is that larger resections may be associated with a higher likelihood of achieving seizure freedom, provided the risks of resecting this tissue is acceptably low^39^. However, this approach may lead to larger-than-necessary resections of more normal cortex or lead to a lower probability of seizure freedom when CD involving eloquent cortex is intentionally preserved^36^. Through a more tailored surgical approach utilizing measures of brain stiffness, we suspect that improved likelihood of seizure freedom can be achieved through more complete resections of CD and, alternatively, the same likelihood of seizure freedom can be obtained in children undergoing smaller cortical resections. Lastly, a novel approach utilizing MRE and brain tonometry to identify areas increased brain stiffness could prevent the need for invasive monitoring with grids, strip and depth electrodes in select cases.

### Strengths and limitations of the digital tonometer

Current technical shortcomings of this device include its limited ability to be used on exposed lateral brain surface and, thus far, not being able to sample the difficult-to-access regions of the brain such as the insular or interhemispheric regions as well as subcortical structures. We are also limited to recording in regions in which the device can be held exactly vertical, a constraint imposed by the requirement that the tonometer’s rod needs to undergo free fall without friction against the walls of the device. Anatomical structures such as the sulci and blood vessels preclude recording from all exposed brain surfaces. Further, the depth of cortex from which the tonometer probe measures stiffness is unknown but most likely limited to a few millimeters. The range of stiffness that is suitable at measuring reliably might be too limited to cover the complete range of brain tissue stiffness that might be encountered. In its current form, the device is cumbersome only yielding point readings. Furthermore, our study is limited by our low patient number and inability to directly correlate our findings with emerging MRE tools. There are also many strengths to using the digital tonometer. Recordings are in real-time, have high spatial resolution and reproducibility. Compared to other intraoperative image-guided neuronavigation methods, this tool is not impacted by brain-shift. Lastly, the components that touch the cortical surface can be sterilized.

## Conclusion

Through this preliminary study, we have demonstrated feasibility of an easy-to-use handheld tool to obtain real-time in vivo measurements of human brain stiffness during open craniotomy. We have identified highly consistent normal values of human brain stiffness across age, gender, ethnicity, and epilepsy substrate within the confines of our patient population. Our early experience demonstrates that underlying MRI lesion, severity of cortical disorganization and recent subdural grid implantation are highly associated with regions of increased brain stiffness. Future multicenter studies are required to determine whether the identification and resection of abnormally stiff areas may result in greater success of epilepsy surgery in patients with CD.
